# Vitamin Supplementation as Possible Prophylactic Treatment against Migraine with Aura and Menstrual Migraine

**DOI:** 10.1155/2015/469529

**Published:** 2015-02-28

**Authors:** Munvar Miya Shaik, Siew Hua Gan

**Affiliations:** Human Genome Centre, School of Medical Sciences, Universiti Sains Malaysia, 16150 Kubang Kerian, Kelantan, Malaysia

## Abstract

Migraine is the most common form of headache disorder globally. The etiology of migraine is multifactorial, with genetic components and environmental interactions considered to be the main causal factors. Some researchers postulate that deficits in mitochondrial energy reserves can cause migraine or an increase in homocysteine levels can lead to migraine attacks; therefore, vitamins could play a vital role in migraine prevention. For instance, riboflavin influences mitochondrial dysfunction and prevents migraine. Genes such as flavoenzyme 5,10-methylenetetrahydrofolate reductase (MTHFR), especially the *C677T* variant, have been associated with elevated plasma levels of homocysteine and migraine with aura. Homocysteine catalyzation requires the presence of vitamins B_6_, B_12_, and folic acid, which can decrease the severity of migraine with aura, making these vitamins potentially useful prophylactic agents for treating migraine with aura. Menstrual migraine, on the other hand, is associated with increased prostaglandin (PG) levels in the endometrium, indicating a role for vitamin E, which is an anti-PG. Vitamin C can also be used as a scavenger of reactive oxygen species for treating neurogenic inflammation in migraine patients. This paper reviews possible therapies based on vitamin supplementation for migraine prophylaxis, focusing on migraine with aura and menstrual migraine.

## 1. Introduction

Migraine is a common, painful, and disabling condition characterized by recurrent, unilateral, and pulsatile attacks of headache that can be moderate to severe in intensity [1]. Most migraine attacks begin at puberty and affect those aged between 35 and 45 years. The prevalence of migraine is higher in women (5% to 25%) than in men (2% to 10%) [2, 3]. The incidence of migraine attacks tends to peak in the middle-aged group but is reported to be lower among adolescents and the elderly (aged above 60 years) [3]. Migraine symptoms vary among individuals, and different symptoms may present during different attacks. Migraine attacks may also differ in length and frequency, usually lasting from 4 to 72 hours. Although individuals may be free of symptoms between attacks, the attacks have an enormous impact on work, family, and social lives.

There are several types of migraines, and they are diagnosed based on the symptoms experienced. Some are triggered by seasonal changes and others by alterations in hormone levels, especially in women. In 1988, the International Headache Society produced a system for classifying migraines, a system also adopted by the World Health Organization [4]. This system was updated in January 2004 [5] and is the established basis for defining types of headaches. The most common forms of migraine fall into two main categories, migraine with aura (MA) and migraine without aura (MO). MO is the most common type of migraine, and approximately 80% of migraine patients are reported to have this type [3]. MA is primarily characterized by the presence of focal neurological symptoms such as blurred vision, vertigo, or hallucination, and these symptoms usually precede or accompany the headache attacks. Some patients also experience a premonitory phase, which occurs hours or days before the headache, followed by a resolution phase. Premonitory and resolution phase symptoms including hyperactivity and hypoactivity, depression, cravings for particular foods, repetitive yawning, and other less typical symptoms are reported by some patients. These symptoms differ from migraine without aura, which is not preceded by aura and may last from 4 to 72 hours. Nearly 31% of migraine patients are reported to have 3 or more headache attacks per month, with 54% of migraine patients reported to have severe impairment in daily functions both at home and at work [3]. Similar prevalence rates have been shown in other populations (European and Asian) with a comparable distribution pattern based on age and sex [6].

It was hypothesized that mitochondria may play a role in the pathogenesis of migraine and the roles of mitochondria disorders and migraine have been well established [7–10]. Mitochondria are small organelles that play a central role in energy generation, reactive oxygen species (ROS) production, regulation of apoptosis, and control of calcium (Ca2+) homeostasis [7]. Since mitochondria play a vital role in the primary mechanism of Ca2+ sequestration in a cell, its dysfunction is postulated to lead to pain hypersensitization [11]. In addition, vasoconstriction during cortical spreading depression (CSD) is caused by increased Ca2+ concentrations within astrocytes. This process is mediated by phospholipase A2 which is an arachidonic acid metabolite [12]. Mitochondria are crucial for the normal functioning of neurons due to their involvement in Ca2+ homeostasis. Therefore, an imbalance of Ca2+ could lead to a range of downstream imbalances, further contributing to increased migraine susceptibility [9].

Cytochrome-c oxidase (COX), also known as complex IV, of the mitochondria respiratory transport chain binds to nitric oxide (NO) [13]. In vessels rich in COX, sequestration of NO may occur, which prevents vasodilatation. COX-negative fibers with increased fat accumulation have also been observed in some patients with migraine [7]. To date, there existed only a single case report of a genetic defect in mitochondria DNA being associated with cluster headache [14]. In addition, two common mitochondria DNA polymorphisms (16519C→T and 3010G→A) have been associated with pediatric cyclic vomiting syndrome and migraine [15].

Oxidative stress has been proposed as a relevant event in the pathogenesis of headaches [16]. Oxidative stress was determined by measuring coenzyme Q10, catalase, and lipid peroxidation (LPO) levels. It is believed that oxidative stress and LPO play a role in the pathogenesis of migraine by regulating cerebral blood flow and energy metabolism both of which may constitute a trigger threshold for migraine attacks [17, 18]. It is known that LPO levels indirectly reflect generation of intracellular ROS as a consequence of oxidative stress as ROS are implicated in pain etiology. Therefore, it is hypothesized that antioxidants (in the form of either vitamins or cofactors) may help to reduce the levels of free radicals which play a vital role in mitochondrial disorders.

Preventive treatment for migraine helps reduce disability by decreasing the frequency, severity, and duration of migraine attacks. The main aim of preventive treatment is to improve the acute treatment response during attacks as well as quality of life (QOL). Preventive treatment is especially crucial for patients with severe migraine attacks and migraine-related disability. Recurring headache-induced disability is a major concern in migraine patients, as it impairs their QOL and ability to work. According to the World Health Organization, migraine is the 19th leading cause of disability in total years lived [6]. Furthermore, migraine is an economic burden to the patient's family as well as their country. In the USA alone, it is estimated that migraine costs approximately USD 1 billion per year in direct medical costs and USD 13 billion per year in lost work productivity [19].

The goal of using a preventive treatment mirrors the criteria for initiating such a treatment. The primary goals of preventive treatment are reduced frequency and severity of migraine headaches, and it is important that these goals be communicated to patients [20–22]. It is also crucial to emphasize early on to patients that migraine has no cure and, therefore, that the goal is to manage the disease and reduce its burden. A patient's disability could be reduced when these main goals are achieved. Other goals of preventive treatment include reducing the use of acute drugs and decreasing visits to the emergency room or the need for surgery. Patients also need to understand that it may take time for a drug to become effective, most often two to three months, even when administered in adequate doses. Asking patients to keep a headache calendar or diary is an effective way to monitor progress.

Medication choices are somewhat limited by their availability in each country. Nonetheless, the first choices of treatment are beta-adrenoceptor blockers (propranolol) [23], calcium channel blockers (flunarizine) [24], anticonvulsants (topiramate) [25], selective serotonin reuptake inhibitors [26], and anticonvulsant drugs (valproic acid) [27]. Beta-adrenoceptor blockers are particularly useful in patients also suffering from hypertension or tachycardia. Although topiramate is widely used as a first-line treatment for episodic and chronic migraine, it leads to weight loss and is also associated with adverse cognitive effects. Valproic acid and flunarizine are also considered to have very good prophylactic properties for migraine, yet valproic acid is often associated with adverse effects and flunarizine is unavailable in many countries, including the United States. According to a Cochrane review [26], selective serotonin reuptake inhibitors for migraine treatment are no more efficacious than placebo. The prophylactic properties of other agents such as magnesium, riboflavin, and coenzyme Q10 are low at best, but their lack of severe adverse effects makes them good alternative treatment options [28–30].

Some studies have shown an association of hyperhomocysteinemia with migraine as well as the roles of vitamins B_6_, B_9_, and B_12_ in lowering the levels of homocysteine in patients with hyperhomocysteinemia [31–35]. There is also evidence that riboflavin (vitamin B_2_) reduces the frequency of migraine attacks [36]. Vitamins also act as antioxidants and work effectively in oxidative stress to slow down the disease progression. This review highlights the roles of several types of vitamins that have the potential to be used as migraine prophylaxis.

## 2. Vitamins

### 2.1. Riboflavin (Vitamin B_2_)

**Figure 1:**
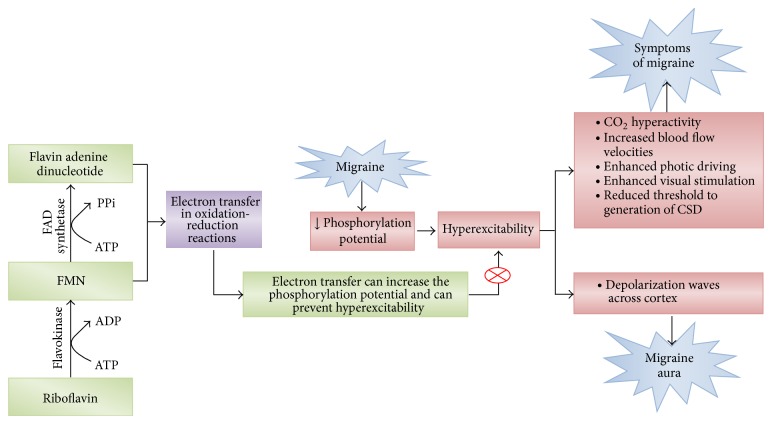
Schematic diagram depicting the possible roles of riboflavin in ameliorating migraine. ATP: adenosine triphosphate, ADP: adenosine diphosphate, FAD: flavin adenine dinucleotide, PPi: pyrophosphate (anion P_2_O_7_^4−^), CO_2_: carbon dioxide, and CSD: cortical spreading depression. Red circled times symbol: inhibition of the pathway.

Riboflavin is a nutrient found in milk, eggs, malted barley, liver, kidney, heart, and leafy vegetables, with yeast considered the richest natural source. Studies have investigated the potential role of riboflavin in preventing migraine symptoms [37]. The interictal reduction of phosphorylation potential in the brain and muscles of migraineurs by riboflavin has previously been reported [38, 39]. The principal forms of riboflavin in tissues and cells are flavin mononucleotide (FMN) and flavin adenine dinucleotide (FAD), which are also coenzymes (Figure 1). These forms of riboflavin are involved in transferring electrons in oxidation-reduction reactions. Patients with mitochondrial encephalomyopathy, lactic acidosis, and stroke-like episodes also show reduced metabolism in mitochondria and experience migraine-like headaches, which have been found to be alleviated by riboflavin [40].

Evidence indicates hyperexcitability in migraine, as shown by carbon dioxide hyperreactivity, elevated cerebral blood flow velocities (observed in transcranial Doppler studies), enhanced photic driving, enhanced visual stimulation, and a reduced threshold for generating CSD. CSD is a characteristic depolarization wave observed in the migrainous cortex that spreads across the cortex to produce migraine aura [41]. Spontaneous electrical discharges are observed during magnetoencephalography as are a lower threshold and increased incidence of phosphenes after transcranial magnetic stimulation [42, 43]. A lower threshold for generating phosphenes is observed even with mild head bumps in migraine patients. These patients also show increased continuous negative variation and abnormal habituation in auditory evoked potentials during electrophysiological studies and increased muscle jitter on electromyography. One characteristic of late migraine attacks is cutaneous hypersensitivity (allodynia and hyperpathia). Further, metabolic studies show an increased cerebral metabolic rate of oxygen consumption and glucose use, reduced phosphorylation, and an inability to quickly respond to increased metabolic demand on magnetic spectroscopy [44]. The abnormalities also extend beyond neural tissues and include mitochondrial dysfunctions in platelets and the production of peripheral markers of oxidative stress (nitric oxide metabolites and thiobarbituric acid-reactive species) [41].

The various clinical trials conducted among migraine patients using vitamin B_2_ are summarized (Table 1). Schoenen et al. (1998) investigated the use of riboflavin (400 mg/day) among migraine patients (*n* = 55) based on the hypothesis that impaired oxygen metabolism may contribute to the development of migraine attacks [36]. The study reported that approximately 59% of migraine patients showed at least 50% of symptom reduction with only minor adverse effects (diarrhea and polyuria) seen. It was also concluded that riboflavin is a good option for migraine prophylaxis due to its high efficacy, excellent tolerability, and low cost. However, in the USA, a randomized, placebo controlled study (*n* = 48) using a combination of high-dose riboflavin (400 mg/day), magnesium (300 mg/day), and feverfew (100 mg/day) found no difference between the high dose (400 mg/day) of riboflavin and a low dose. Nonetheless, both groups of patients who received either low or high doses of riboflavin had a significant reduction in the number of migraine attacks and the number of days with migraine [45].

A study among Australian children (*n* = 48) reported that the administration of high doses of riboflavin (at least 200 mg/day) did not produce any significant effect in ameliorating migraine when compared to placebo [46]. A study conducted in Italian pediatric and adolescent migraine patients (*n* = 41), however, suggested that riboflavin (200 or 400 mg/day) may be effective in treating migraine [47]. This study suggested that the 200 mg/day dose of riboflavin is sufficient for treating migraine when compared to the 400 mg/day dose, even though the latter dose is recommended for patients with severe migraine [47]. The study reported that 68.4% of migraine patients had a 50% or greater reduction in the frequency of migraine attacks and 21.0% in its intensity, with only 5% of patients experiencing minor adverse effects such as vomiting and appetite changes.

A randomized, double blind, placebo controlled, crossover clinical trial of riboflavin conducted among Dutch children (*n* = 42; 6–13 years) with migraine administered a medium dose range (approximately 50 mg/day). The study found no significant difference between migraine frequency and the severity or duration of migraine between subjects who received riboflavin or placebo. Therefore, the researchers concluded that there is insufficient evidence for the clinical use of 50 mg of riboflavin as migraine prophylaxis [48]. However, Boehnke et al. (2004) demonstrated the efficacy of riboflavin (400 mg/day) among migraine patients (*n* = 23) coming from a tertiary clinic [37]. The study also reported a reduction (*P* < 0.05) in the headache frequency from 4 days/month at baseline to only 2 days/month following riboflavin use but there was no change in either the duration or the intensity of headache experienced.

Due to the conflicting evidence available, more studies, especially randomized clinical trials, are required to conclusively define the exact role of riboflavin as a possible prophylactic agent for migraine. The different race, age, and gender of the subjects involved in various studies add to the variabilities further compounded by the fact that migraine is contributed by multifactorial factors.

### 2.2. Vitamins B_6_, B_9_, and B_12_

The flavoenzyme 5,10-methylenetetrahydrofolate reductase (MTHFR) regulates the flow of folate (vitamin B_9_) between the production of nucleotides and the supply of methyl groups during methionine synthesis [49, 50] and has major effects on the distribution of intracellular folate [51].* MTHFR* also plays a pivotal role in the pathogenesis of migraine and hyperhomocysteinemia. The MTHFR enzyme encoded by MTHFR catalyzes the reduction of 5,10-methylenetetrahydrofolate to 5-methyltetrahydrofolate, an ethyl group donor to the intermediary metabolite homocysteine during its metabolism to methionine. Methionine is used in the synthesis of the universal methyl donor S-adenosylmethionine. Upon donation of the methyl group, S-adenosylmethionine is converted to S-adenosylhomocysteine, which is subsequently hydrolyzed to homocysteine and adenosine [52].

**Figure 2:**
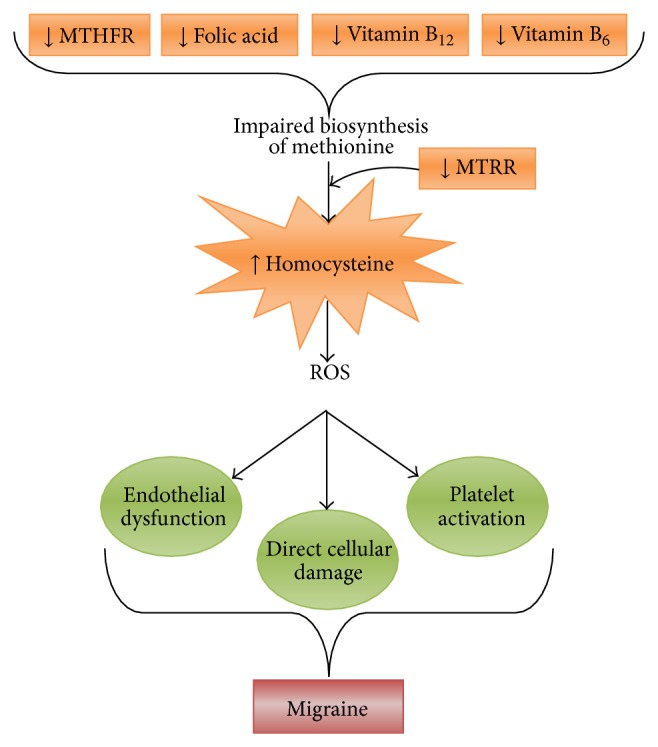
Schematic representation depicting the role of vitamins B_6_, B_12_, and folic acid in migraine pathophysiology. MTRR (or MSR): methionine synthase reductase, MTHFR: methylene tetrahydrofolate reductase, and ROS: reactive oxygen species.

Hyperhomocysteinemia is caused by abnormal methionine biosynthesis due to deficiencies in folate, vitamin B_12_, and vitamin B_6_ [53]. Folate is also needed to drive the methionine synthesis pathway, as a lack of dietary folate and/or reduced MTHFR enzymatic activity can result in increased homocysteine levels in blood plasma [54] (Figure 1). This relationship makes homocysteine a key intermediate in one-carbon metabolism and further clarifies the involvement of B vitamins in transferring one-carbon units and their relation to the plasma concentrations of total homocysteine (Figure 2) [55, 56].

Table 2 describes the characteristics of various clinical trials conducted among migraine patients using vitamins B_6_, B_9_, and B_12_. A clinical study conducted among Italian children with migraine [32] reported that folic acid (5 mg/daily) supplementation for six months led to a significant reduction (52%) in homocysteine levels during treatment follow-up. However, the study reported no statistically significant effects of folic acid on reducing migraine attacks when the plasma levels of homocysteine were compared at the beginning and end of the treatment.

Lea et al. (2009) reported that vitamin supplementation (2 mg of folic acid, 25 mg of vitamin B_6_, and 400 *μ*g of vitamin B_12_) in MA patients reduced homocysteine levels by 39% when compared to baseline, and the effect was significantly greater than with placebo. Vitamin supplementation also significantly reduced the prevalence of migraine disability from 60% at baseline to 30% after 6 months, whereas no reduction was observed in the placebo group. The headache frequency and pain severity were also significantly reduced, with no reductions found in the placebo group. In this patient group, the effect of the treatment on both homocysteine levels and migraine disability was associated with the* MTHFR C677T* genotype, whereby carriers of the C allele had a better response to treatment than those with the TT genotype. Therefore, the researchers concluded that lowering homocysteine levels using vitamin supplementation reduces migraine disability in a subgroup of migraine patients [31], particularly in patients with MA.

A study conducted on Australian female Caucasians with MA [35] also reported that vitamin (B_6_, B_9_, and B_12_) supplementation not only significantly reduced homocysteine levels but also reduced the severity of migraine headache and disability among migraineurs when compared to placebo. When the vitamin-treated group was stratified based on genotype, carriers of the C allele of the* MTHFR C677T* variant showed significantly greater reductions in homocysteine levels, the severity of migraine pain, and the percentage of disability when compared with those with the TT genotype. Similarly, the A allele carriers of the methionine synthase reductase (*MTRR A66G*) variants also showed significantly greater reductions in homocysteine levels, the severity of pain in migraine, and the percentage of migraine disability when compared with those with the GG genotypes. However, despite the genotypic influences of homocysteine levels, the therapeutic efficacy of vitamins B_6_, B_9_, and B_12_ in patients with the* MTRR* variant was found to be independent of the* MTHFR* variant. Further studies are required to elucidate the exact mechanism of interaction between the genetic variants when vitamins are given as prophylaxis to MA patients.

### 2.3. Vitamin E

**Figure 3:**
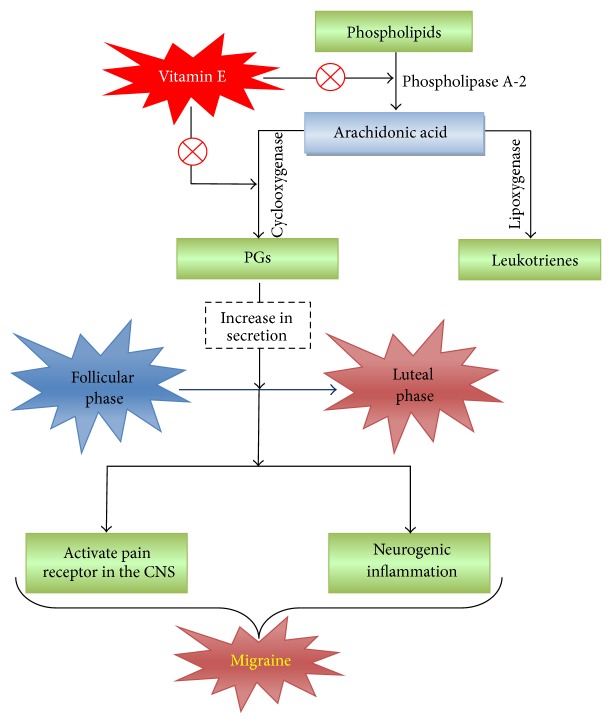
Schematic representation of the possible roles of vitamin E in relation to PGs as prophylaxis of menstrual migraine. CNS: central nervous system and PG: prostaglandins. Red circled times symbol: inhibition of the pathway.

Numerous mechanisms have been proposed for menstrual migraine, including a reduction in magnesium levels, platelet dysfunction, central serotonin dysmodulation, and prostaglandin (PG) release [57, 58]. For example, it has been reported that there is a threefold increase in PG levels in the endometrium between the follicular and luteal phases, which increases further during menstruation (Figure 3). The high levels of PG found in the serum during the premenstrual phase led to the use of drugs with anti-PG effects [59]. Vitamin E inhibits the release of arachidonic acid and the conversion of arachidonic acid to PG by acting on the enzymes phospholipase A2 and cyclooxygenase [60].

Vitamin E, which is an anti-PG agent with a reportedly low side effect profile, can effectively relieve headache pain and associated migraine symptoms [61, 62]. It also reduced functional disability and the need for rescue medications [61, 62]. A limitation of current treatment strategies is that even though a short-term use of prophylactic agents such as nonsteroidal anti-inflammatory drugs and triptans can ameliorate headaches, many patients tend to experience worsening of headache symptoms after the short-term treatment is stopped. However, with the use of vitamin E therapy (400 IU/daily for five days during menstruation for 3 cycles), there have been no reported breakthrough headaches. Vitamin E may provide a useful treatment option for women suffering from this debilitating and refractory migraine subtype [61, 62]. Ziaei et al. reported that there was a reduction in the pain severity and improvement in the functional disability scales among female migraine patients who used vitamin E for five days during their menstruation periods [61].

Therefore, further studies conducted in a large population could help in determining the efficacy of vitamin E in the treatment of women who consistently experience menstrual migraine.

### 2.4. Vitamin C

Epidemiological studies have established an increased risk of developing complex regional pain syndrome (CRPS, a painful neuroinflammatory disorder of the limbs due to tissue or nerve damage) among migraineurs and patients with asthma and inflammatory bowel disease [63]. The link between these seemingly disparate medical conditions may be a shared pathophysiology involving neurogenic inflammation, in which the release of neuropeptides such as substance P (SP) and calcitonin gene-related peptide (CGRP) produces reactive oxygen species (ROS) that in turn induce tissue damage and microvascular dysfunction [64]. In support of this theory, increased levels of SP, CGRP, and ROS have been found in both CRPS and migraine patients [65, 66]. Administration of vitamin C, which is a ROS scavenger and is an antioxidant, in doses of 200–1,500 mg daily for up to 50 days following wrist and ankle injuries was shown to significantly reduce the incidence of CRPS and has been proposed as a preventive therapy for this condition [67–71]. It is thought that vitamin C “mops up” ROS produced during neurogenic inflammation in the early stages of CRPS. Based on this model, it seems logical to assume that vitamin C may also modulate the effects of neurogenic inflammation and ROS in migraine.

To date, the efficacy of vitamin C as a prophylactic treatment for migraine has not been investigated in a randomized controlled trial (RCT). A small nonrandomized trial using a combination of antioxidants and an RCT using the antioxidant vitamin E reported significantly improved migraine outcomes [61, 72]. In conclusion, migraine may be considered a “CRPS of the brain,” with neurogenic inflammation and ROS generation as common mechanisms. Because vitamin C significantly reduces the risk of CRPS after injury, it may also act as a prophylactic agent against migraine. An RCT investigating the hypothesis that the administration of 1,000 mg of vitamin C daily will reduce the incidence and severity of migraine attacks is urgently needed, as vitamin C is a cheaper alternative and has relatively fewer side effects than other drugs used for migraine treatment.

## 3. Conclusion

Vitamins are useful for migraine prevention, and riboflavin is reported to be an effective alternative prophylactic agent among pediatric and adult migraine patients by increasing the synthesis of FMN and FAD to generate phosphorylation potentials. Lowering homocysteine levels through vitamin supplementation, specifically with folic acid and vitamins B_6_ and B_12_, may reduce migraine disability in patients with MA. However, a larger clinical trial is warranted to establish whether vitamin supplementation using folic acid is a safe, inexpensive, and effective preventative treatment for increasing the quality of life of migraineurs and to determine whether such treatment should be based on the* MTHFR* genotype. Although vitamin E has the potential to be used in the treatment of women suffering from menstrual migraine, a debilitating and refractory migraine subtype, further large population studies are needed to determine its efficacy. Additionally, the potential of vitamin C to be used as an ROS scavenger for neurogenic inflammation in migraine patients needs to be confirmed in RCTs.

## Figures and Tables

**Table 1:** Clinical trials conducted among migraine patients using riboflavin (vitamin B_2_).

Study location	Control		Placebo		Duration of administration	Outcomes	*P* value	Reference
	Dose	Sample size	Dose	Sample size				
Belgium	B_2_ (400 mg/day)	55	Placebo	55	3 months	59% of reduction in frequencies of migraine attack and days with headache	0.0120	[36]

USA	B_2_ (400 mg/day) + magnesium = (300 mg/day) + feverfew (100 mg/day)	24	25 mg/day of vitamin B_2_	24	3 months	50% or greater reduction in migraine in 40% of migraine patients	0.0006	
						Days with migraine	0.0700	[45]
						Migraine index	0.0006	

USA	B_2_ (400 mg/day) + magnesium = (300 mg/day) + feverfew (100 mg/day)	24	25 mg/day of vitamin B_2_	24	1 month	50% or greater in reduction of migraine in 33% of migraine patients	<0.0001	
						Days with migraine	0.0400	[45]
						Migraine index	0.1000	

Australia	B_2_ = 200 mg/day	21	Placebo	27	1 month	50% or greater reduction in headaches	0.1250	[46]

Italy	B_2_ (200 mg/day or 400 mg/day) randomly administered	41	Compared with baseline		6 months	50% or greater reduction in headache frequency among 68.4% of patients 50% or greater reduction in intensity of headache	<0.010	[47]

Netherlands	B_2_ (50 mg/day)	42	Placebo	42		Reduction in frequency of migraine attacks	0.4400	[48]

Germany	B_2_ (400 mg/day)	23	Compared with baseline		3 months	Frequency of headache	<0.0010	
						Duration of attacks	0.0980	[37]
						Headache intensity	0.2960	

Germany	B_2_ (400 mg/day)	23	Compared with baseline		6 months	Frequency of headache	0.0050	
						Duration of attacks	0.0980	[37]
						Headache intensity	0.2960	

**Table 2:** Characteristics of clinical trials conducted among migraine patients using vitamins B_6_, B_9_, and B_12_.

Study location	Control		Placebo	Duration of administration	Outcomes	*P* value	Reference
	Dose	Sample size	Dose Sample size				
Australia	Vitapop tablet containing B_6_ (25 mg), B_9_ (2 mg) & B_12_ (400 *μ*g) once daily	37	Compared with baseline	6 months	Migraine disability	0.010	
					Frequency of headache	0.040	[36]
					Headache pain severity	0.002	

Australia	Vitapop tablet containing B_6_ (25 mg), B_9_ (2 mg) & B_12_ (400 *μ*g) once daily	103	Compared with baseline	6 months	Migraine disability	0.022	
					Frequency of headache	0.460	[45]
					Headache pain severity	0.017	

## Abbreviations

CGRP: Calcitonin gene-related peptide
CRPS: Complex regional pain syndrome
CSD: Cortical spreading depression
FAD: Flavin adenine dinucleotide
FMN: Flavin mononucleotide
MTHFR: 5,10-Methylenetetrahydrofolate reductase
MTRR: Methionine synthase reductase
MA: Migraine with aura
MO: Migraine without aura
PG: Prostaglandins
QOL: Quality of life
RCT: Randomized controlled trials
ROS: Reactive oxygen species
SP: Substance P.
